# Under diagnosis of intestinal schistosomiasis in a referral hospital, North Ethiopia

**DOI:** 10.1186/s13104-018-3355-0

**Published:** 2018-04-16

**Authors:** Megbaru Alemu, Eyob Zigta, Awoke Derbie

**Affiliations:** 1 Department of Microbiology, Immunology and Parasitology, Bahir Dar University, Po. Box 79, Bahir Dar, Ethiopia; 2 Department of Medical Parasitology and Vector Biology, Mekelle University, Mekelle, Ethiopia

**Keywords:** Wet mount, Formol ether concentration, Kato-Katz, Mekelle, Ethiopia

## Abstract

**Objective:**

The present cross-sectional study was aimed at determining the magnitude of under diagnosis of intestinal schistosomiasis among patients requested for routine ova/parasite examination at Ayder referral hospital.

**Results:**

A total of 280 stool samples were collected and only 5% of the patients were positive for ova of *Schistosoma mansoni* in the routine direct wet mount microscopy. On the other hand, 12.5% of the patients were positive for ova *Schistosoma mansoni* when the stool samples were processed by either Kato Kat or formol ether concentration techniques. Moderate test agreement (κ = 0.48) was recorded for wet mount. Formol-ether concentration (κ = 0.89) and Kato-Katz (κ = 0.92) showed excellent agreements with the ‘Gold’ standard. Direct wet mount technique exhibited the poorest sensitivity (35%) of detection of ova of *Schistosoma mansoni*. Hence, the Kato-Katz technique should be implemented in parallel with the direct wet mount microscopy for *Schistosoma mansoni* presumptive patients.

## Introduction

Human bilharziasis is caused by the trematode species of *Schistosoma mansoni*, *Schistosoma hematobium*, *Schistosoma japonicum*, *Schistosoma intercalatum* and *Schistosoma mekongi* [1]. The disease is highly prevalent throughout Africa, South America and several Caribbean islands [2]. It is one of the most widespread of all human parasitic diseases, ranking second only to malaria in terms of its socioeconomic and public health importance in tropical and subtropical areas [3]. Estimates suggest that over 250 million people were infected and the disease caused 11,700 deaths and a global burden of 3.3 million disability-adjusted life years [4].

In Ethiopia, schistosomiasis is widely spread in the country where endemic areas are located in the altitudinal range of 1200–2000 m above sea level [5]. Rapid spread of the disease also appears to have been facilitated in areas which were originally non-endemic as a result of the initiation of water-based development schemes [6]. High infection rates of *S. mansoni* were reported in the hyper endemic areas of northwestern, northeastern and northern parts of the country [6–8].

Examination of stool is the primary method of diagnosing suspected *Schistosoma mansoni* infections. There are several diagnostic techniques such as Kato-Katz, wet-mount, and formol-ether concentration technique (FECT). Despite its low sensitivity, the direct wet mount is the only method employed for diagnostic purpose of intestinal parasites in general and schistosomiasis in particular in health institutions of Ethiopia while FECT and Kato-Katz are reserved for research purpose [9–12]. The Kato-Katz method is characteristically rapid, easy to perform and require minimal training. The formol-ether concentration technique on the other hand is time-consuming, and requires several materials.

Schistosome species lay only few numbers of eggs and only two-third of the eggs are excreted intermittently with stool, making single wet mount microscopy prone for false negative results [13].

The reliable diagnosis of intestinal schistosomiasis therefore requires a more rapid, economical, easy, and sensitive method. Stool examination for intestinal parasitosis is performed solely by wet mount procedure at Ayder referral hospital. It has been a custom that clinicians sending stool specimens of patients with presumptive *S. mansoni* infection to Microbiology and parasitology research laboratory whose stool examination results turned negative in Ayder hospital laboratory. Consequently, ova of *S. mansoni* were isolated from most of the referred stool specimens in the research laboratory. Hence, we sought to assess performance of wet mount against the Kato-Katz and FECT in the diagnosis of intestinal schistosomiasis and to recommend the best technique in the hospital.

## Main text

### Methods

#### Study design and area

This cross-sectional study was conducted in Ayder referral hospital, North Ethiopia, from August to October 2016. The Hospital provides referral and non-referral services to more than 8 million populations in its catchment areas. It provides a broad range of medical services to both in and outpatients of all age groups. With the total capacity of about 500 inpatient beds in four major departments and other specialty units, the hospital is also used as a teaching hospital for the College of Health Sciences, Mekelle University. Stool examination for intestinal parasitic infections in general and intestinal schistosomiasis in particular is mainly based on direct wet mount microscopy.

#### Sample collection and parasitological examination

Patients were provided with stool cups to bring adequate stool samples. The routine direct wet mount microscopy performed in the hospital laboratory. The Kato-Katz and formol-ether concentration (FEC) methods, on the other hand, were performed in microbiology and parasitology research laboratory.

The test procedures were carried out in accordance with standard protocols reported by World Health Organization (WHO) [14].

##### Kato-Katz method

An approximately 42 mg fecal sample was sieved through a 200 µm Kato nylon screen mesh. The stool was transferred into a 6 mm hole of a template on a microscopic slide and covered with glycerol soaked cellophane strip. The microscopic examination then proceeded to identify schistosome eggs and to calculate the number of eggs per gram (EPG) of feces [15]. Based on egg counts, cut-off values for classification of the intensity of infection were used. The intensity of *S. mansoni* was classified into: light infection (1–99 EPG), moderate (100–399 EPG) and heavy (≥ 400 EPG) [14].

##### Formol ether concentration technique (FEC)

Approximately 500 mg of feces was mixed with 10 ml of normal saline and the mixed stool was strained via gauze into a funnel. The strained contents were collected in a centrifuge tube. About 2.5 ml of 10% formaldehyde (Loba Chemie Pvt Ltd., 107, Wodehouse Road, Jehangir villa, Mumbai-40005, India) and 1 ml of diethyl ether (Blulux laboratories Pvt Ltd. 121005) was then added and centrifuged at 1000*g* for 3 min. The supernatant was removed and a drop of the sediment was covered with cover glass for a microscopic investigation [16].

##### Wet mount preparation

Fresh stool samples (approximately 2 mg of stool) were put on a slide with wooden applicator, emulsified with a drop of physiological saline (0.85%) covered with a cover slide and examined at 10× and 40× microscopic objectives [16].

#### Data entry and analysis

Data were entered and analyzed using SPSS version 20 statistical software. Estimation of the performance of the three diagnostic tests was made by taking the combined results of the wet mount, FEC and Kato-Katz tests as a “Gold” standard diagnostic test, because stool investigation for intestinal parasitosis lacks ‘Gold’ standard method [17].

Sensitivity, specificity, PPV (positive predictive value), NPV (negative predictive value) and Kappa value of wet mount, FEC and Kato-Katz techniques were computed against the ‘Gold’ standard. The kappa score was used to estimate the agreement between stool diagnostic tests and the ‘Gold’ standard.

#### Data quality control

Laboratory technicians in charge of microscopic investigations were blinded to the results of the wet mount, FECT, and Kato-Katz. A different laboratory technician was responsible for preparing and reading Kato-Katz and FEC. In addition, results of the wet mount, Kato-Katz, and FEC techniques were recorded on different sheets to ensure strict blinding.

#### Results

The overall prevalence of *S. mansoni* infection was 40 (14.3%) with a parasitic load ranging from 24 to 480 eggs per gram of stool. *S. mansoni* infections were predominantly light, 65% and moderate 35%. The peak prevalence of *S. mansoni* infection was recorded for the 10–14 years of age (17.6%) followed by those 15 and above years of age (14.5%). Mean intensity of infection was also higher in the age group 10–14 years (224 EPG). The overall prevalence of infection was 12.7% for females and 15.8% for male participants (P > 0.05). The mean intensity of infection was also higher for males (201 EPG) than females (169 EPG) (Table 1).

**Table 1:** Prevalence and intensity of *S. mansoni* with age and sex at Ayder referral hospital, 2016

	*S. mansoni* infection			Total n (%)
	Positive n (%)	Negative n (%)	Mean intensity (EPG)	
Gender				
Male	23 (15.8)	123 (84.2)	201	146 (100)
Female	17 (12.7)	117 (87.3)	169	134 (100)
Age (years)				
5–9	4 (9.3%)	39 (90.7%)	203	43 (100)
10–14	9 (17.6)	42 (82.4)	224	51 (100)
≥ 15	27 (14.5)	159 (85.5)	168	186 (100)

The prevalence of *S. mansoni* using wet mount, Kato-Katz and FEC was 5, 12.5 and 12.5%, respectively (Table 2). The detection rate when two techniques were used at a time was 13.2% for wet mount and Kato-Katz, 12.9% for wet mount and FEC and 13.6% for Kato-Katz and FEC. The detection rate was 14.3% when all the three tests were used together (Table 2). Kato-Katz detected 23 samples that were negative by wet mount. Similarly, 21 samples were detected by FEC that were negative by wet mount technique.

**Table 2:** Prevalence of *S. mansoni* identified in stool diagnostic tests at Ayder referral hospital, 2016

Diagnostic methods	No examined	Positive n (%)	Negative n (%)
Wet mount	280	14 (5.0)	266 (95)
Kato-Katz	280	35 (12.5)	245 (87.5)
FEC	280	35 (12.5)	245 (87.5)
Kato-Katz + wet mount	280	37 (13.2)	243 (95)
FEC + wet mount	280	36 (12.9)	244 (87.1)
Kato-Katz + FEC	280	38 (13.6)	242 (86.4)
KK + FEC + WM	280	40 (14.3)	240 (85.7)

The sensitivity of wet mount, Kato-Katz and FEC in the detection of *S. mansoni* infection was 35 (95% CI 20.6–51.7), 87.5 (95% CI 73.2–95.8), 85 (95% CI 70.2–94.3), respectively. Similarly, NPV of 90.2 (95% CI 88–92.1), 98 (95% CI 95.5–99.1) and 97.6 (95% CI 95–98.8) were reported, respectively for the wet mount, FEC, and Kato-Katz. Moderate test agreement (κ = 0.48) was recorded for the wet mount. FEC (κ = 0.89) and Kato-Katz (κ = 0.92) on the other hand showed excellent agreements with the Gold standard (Table 3).

**Table 3:** The performance of stool diagnostic techniques for diagnosis of intestinal schistosomiasis at Ayder referral hospital, 2016

Diagnostic methods	Gold standard				
	Sensitivity (95% CI)	Specificity (95% CI)	PPV (95% CI)	NPV (95% CI)	Kappa value
Wet mount	35 (20.6–51.7)	100	100	90.2 (88–92.1)	0.48
Kato-Katz	87.5 (73.2–95.8)	100 (98.5–100)	100	98 (95.5–99.1)	0.92
FEC	85 (70.2–94.3)	99.6 (97.7–99.9)	97.1 (82.7–99.6)	97.6 (95–98.8)	0.89

### Discussion

The prevalence of *S. mansoni* infection in this study was 14.3%, which was higher than previous reports [18, 19]. This might be explained in part by the nature of the study participants, as symptomatic patients were recruited in our study. The prevalence and intensity of *S. mansoni* were found to be higher in males than females in the current study. This goes in agreement with other studies in Africa [20–22]. The existence of more outdoor activities and water exposure habits among males might have contributed to these findings. The peak prevalence of *S. mansoni* was recorded for the 10–14 age group which was consistent with similar other findings [18].

Diagnosis of intestinal parasitosis is based on detection of the eggs in stool samples examined through a variety of parasitologic methods, and no single technique is satisfactory. In Ethiopia, healthcare laboratories employ the direct wet mount as the preferred stool parasitological examination technique by virtue of its simplicity, low cost, and rapidity.

Although the wet mount technique is the preferred method in resource-limited settings [23], reliance on it as the sole diagnostic tool in routine practice is very likely to cause misdiagnosis of infections. This, in turn, leads to grave clinical and public health consequences [24].

It is supported by our study in which wet mount technique detected only one-third (14/40) of the study participants infected by *S. mansoni*.

The Kato-Katz method is characteristically rapid, easy to perform and require minimal training. Its high sensitivity (87.5%) and specificity (100%) for the diagnosis of *S. mansoni* compared to the wet mount is well noted in our study. It was also slightly sensitive than formol-ether concentration. Consistent results were reported by others [17, 25]. The technique is a useful tool for the quantification of egg counts to determine infection intensities. These qualities make the Kato-Katz the most frequently employed method in research works [14].

Time-consuming procedures, the requirement of trained personnel and several materials/equipment make the FEC technique the most expensive method to be considered as an alternative for routine laboratory diagnosis of intestinal schistosomiasis. However, our study revealed that the sensitivity and specificity of FEC are not much less than the Kato-Katz method for detecting eggs of *S. mansoni*.

The prevalence of *S. mansonia* via wet mount, FEC, and Kato-Katz was 5, 12.5 and 12.5%, respectively. Our study revealed that the Kato-Katz and FEC techniques had about threefold increased in the detection rate of *S. mansoni* than the wet mount. Consistent findings were reported in previous studies [17, 25]. Our study revealed that Kato-Katz (87.5%) was slightly sensitive than FEC (85%) to detect *S. mansoni*. Similarly, Kato-Katz showed the highest agreement with the Gold standard (κ = 0.92) while wet mount showed the lowest agreement (κ = 0.48). This showed that the use of Kato-Katz can ultimately reduce misdiagnosis of intestinal schistosomiasis, and reduce morbidity and mortality due to schistosomiasis. This was supported by other findings in Ethiopia [17, 25].

### Conclusion

Direct wet mount technique exhibited the poorest sensitivity of detection of ova of *Schistosoma mansoni*. Most of the infections were predominantly light in the study area which requires implementation of concentration methods. Hence, the Kato-Katz technique should be implemented in parallel with the direct wet mount microscopy for *Schistosoma mansoni* presumptive patients.

## Limitations of the study

In this study, we collected only a single stool sample and hence we were unable to describe the variation of egg counts as *Schistosoma mansoni* female worms undergo intermittent egg excretion. Future investigations should focus on the feasibility of the concentration techniques in terms of the turn-around-time (TAT) in health facilities that serve large number of outpatients.

## Abbreviations

FECT: formol ether concentration techniques
EPG: eggs per gram
WHO: World Health Organization
PPV: positive predictive value
NPV: negative predictive value

## References

[1] Steinmann P, Keiser J, Bos R, Tanner M, Utzinger J (2006). Schistosomiasis and water resources development: systematic review, meta-analysis and estimates of people at risk. Lancet Infect Dis.

[2] Gryseels B, Polman K, Clerinx J, Kestens L (2006). Human schistosomiasis. Lancet.

[3] Colley DG, Bustinduy AL, Secor WE, King CH (2014). Human schistosomiasis. Lancet.

[4] Murray CJL, Vos T, Lozano R, Naghavi M, Flaxman AD, Michuad C (2012). Disability-adjusted life years (DALYs) for 291 diseases and injuries in 21 regions, 1990–2010: a systematic analysis for global burden disease study 2010. Lancet.

[5] Erko B, Abebe F, Berhe N (2002). Control of *Schistosoma mansoni* by the soapberry Endod (*Phytolacca dodecandra*) in Wollo, North eastern Ethiopia post-intervention prevalence. East Afr Med J.

[6] Tiruneh M, Fantahun M, Kassu A, Tiruneh G, VanLieshout L (2001). *Schistosomiasis mansoni* in school attenders and non-attenders in Northwest Ethiopia. Ethiop J Health Dev.

[7] Birrie H, Abebe F, Gundersen SG, Medhin G, Berhe N, Gemetchu T (1998). Epidemiology of *Schistosomiasis mansoni* in three endemic communities in north-east Ethiopia: baseline characteristics before endod based intervention. Ethiop Med J.

[8] Birrie H, Woldemichael T, Redda A, Chane T (1994). The status of *Schistosoma mansoni* and snail hosts in Tigray and northern Wello regions, northern Ethiopia. Ethiop Med J.

[9] Utzinger J, Rinaldi L, Lohourignon LK (2008). FLOTAC: a new sensitive technique for the diagnosis of hookworm infections in humans. Trans R Soc Trop Med Hyg.

[10] Kassahun H, Abrham D, Yemane Y, Birhanu E (2011). Comparision of the Kato katz and FLOTAC techniques for the diagnosis of soil-transmitted helminth infections. Parasitol Int.

[11] Knopp S, Rinaldi L, Khamis IS (2009). A single FLOTAC is more sensitive than triplicate Kato-Katz for the diagnosis of low-intensity soil-transmitted helminth infections. Trans R Soc Trop Med Hyg.

[12] Tarafder MR, Carabin H, Joseph L, Balolong E, Olveda R, McGarvey ST (2010). Estimating the sensitivity and specificity of Kato-Katz stool examination technique for detection of hookworms, *Ascaris lumbricoides* and *Trichuris trichiura* infections in humans in the absence of a ‘gold standard’. Int J Para.

[13] Gillespie SH, Pearson RD (2001). Principles and practice of clinical parasitology.

[14] World Health Organization (1991). Basic laboratory methods in medical parasitology.

[15] World Health Organization (2002). Prevention and control of schistosomiasis and soil-transmitted helminthiasis.

[16] Cheesbrough M (2009). District laboratory practice in tropical countries part 1.

[17] Yimer M, Hailu T, Mulu W, Abera B (2015). Evaluation performance of diagnostic methods of intestinal parasitosis in school-age children in Ethiopia. BMC Res Notes.

[18] Alemu M, Hailu A, Bugssa G (2014). Prevalence of intestinal schistosomiasis and soil-transmitted helminthiasis among primary school children in Umolante district, South Ethiopia. Clin Med Res.

[19] Girum T (2005). The prevalence of intestinal helminthic infections and associated risk factors among school children in Babile town, eastern Ethiopia. Ethiop J Health Dev.

[20] Alembrhan A, Tadesse D, Zewdneh T (2013). Infection prevalence of *Schistosoma mansoni* and associated risk factors among school children in suburbs of Mekelle city, Tigray, northern Ethiopia. MEJS.

[21] Mengistu L, Berhanu E (2004). Prevalence of intestinal parasites among schoolchildren in a rural area close to the southeast of Lake Langano, Ethiopia. Ethiop J Health Dev.

[22] Okpala H, Agwu I, Chimezie O, Nwobu G, Ohihoin A (2004). A survey of the prevalence of schistosomiasis among pupils in Apata and Laranto areas in Jos, Plateau State. OJHAS.

[23] Wirkom V, Tata R, Agba M, Nwobu G, Ndze R, Onoja O, Utien G, Bongkisheri L, Nsadzetreno V, Banseka E (2007). Formol-petrol stool concentration method: a cheap novel technique for detecting intestinal parasites in resource-limited countries. IJTM.

[24] Barnabas MM, Aboi JKM (2005). Missed diagnosis of schistosomiasis leading to unnecessary surgical procedures in Jos university teaching hospital. Trop Dr.

[25] Endris M, Tekeste Z, Lemma W, Kassu A (2013). Comparison of the Kato-Katz, wet mount and formol-ether concentration diagnostic techniques for intestinal helminth infections in Ethiopia. ISRN Parasitol.

